# Do People with Schizophrenia Lack Emotional Intelligence?

**DOI:** 10.1155/2012/495174

**Published:** 2012-12-13

**Authors:** Sara Dawson, Lisa Kettler, Cassandra Burton, Cherrie Galletly

**Affiliations:** ^1^School of Psychology, The University of Adelaide, Adelaide, SA 5000, Australia; ^2^The Adelaide Clinic, Ramsay Health Care (SA) Mental Health Services, Gilberton, SA 5000, Australia; ^3^Discipline of Psychiatry, School of Medicine, The University of Adelaide, Adelaide, SA 5000, Australia; ^4^Northern Mental Health, Adelaide Metro Mental Health Directorate, Adelaide, SA 5000, Australia; ^5^The Adelaide Clinic Consulting Suites, 33 Park Terrace, Gilberton, SA 5081, Australia

## Abstract

Social cognition is a domain of cognitive function that includes the ability to understand and manage social interactions. Emotional intelligence (EI) has been identified as a component of social cognition and is defined as the ability to identify, use, understand, and manage emotions. Neurocognitive impairments are known to be associated with poorer social function in people with schizophrenia, but less is known about the relationships between EI, neurocognition, and social function. The current study assessed EI using the Mayer-Salovey-Caruso Emotional Intelligence Test (MSCEIT) in 20 people with schizophrenia and 20 controls. The schizophrenia group had significantly lower scores on all measures of EI and demonstrated poorer neurocognition and social functioning than controls. The difference between schizophrenia and control groups was greatest for the Understanding Emotions Branch of the MSCEIT. The neurocognition score and total EI score accounted for 18.3% of the variance in social function in the control group and 9.1% of the variance in social function in the schizophrenia group. Our results suggest that a total EI score is not a useful predictor of overall social function and it may be more clinically useful to develop an individual profile of social cognitive abilities, including EI, to form a remediation program.

## 1. Introduction

Social cognition refers to aspects of cognition that are critical for social functioning and interpersonal relationships, such as accurate perception of the emotions of others, the ability to recognize important social cues, theory of mind (TOM), and the management of emotions in social situations [1]. Various components of social cognition have been found to be impaired in people with schizophrenia [2, 3]. Impaired social cognition may also be a risk factor for psychosis—Kim et al. [4] reported that poor performance on social cognition tasks was associated with higher rates of transition to psychosis in young people who were at ultra-high risk of psychosis.

Emotional intelligence (EI) is an important component of social cognition [5, 6]. EI is defined as the ability to process, understand, and manage emotions. EI is most commonly assessed using the Mayer-Salovey-Caruso Emotional Intelligence Test, Version 2.0 (MSCEIT) [7, 8]. Mayer and Salovey [6] identified four components (termed Branches) of EI. The four Branches are Identifying Emotions (the perception of emotion in the self and others); Facilitating or Using Emotions (the capacity to use and generate emotions, and to use emotions in cognitive tasks such as problem-solving and creativity); Understanding Emotions (the ability to comprehend complex information about emotions, including changes or degrees of emotion); and Managing Emotions (the ability to regulate emotions in ourselves and others, and use this information effectively in social interactions). 

The MSCEIT has been widely used in normal populations and has been shown to have good face and content validity [8]. Factor analysis, utilising data from almost 2000 subjects, strongly supports the four-branch model of EI [8]. Higher levels of EI have been shown to be associated with better social engagement and quality of social interaction [9]. People with higher levels of EI are less likely to engage in aggressive behaviour and are less likely to use tobacco or drink excessively [10, 11]. Several studies have demonstrated small but positive correlations between EI and general intelligence [7, 12]. These findings suggest that EI is a useful construct that is reliably associated with real world functioning in normal subjects.

The National Institute of Mental Health Measurement and Treatment Research to Improve Cognition in Schizophrenia (MATRICS) initiative evaluated a number of tests of social cognition and selected the Managing Emotions branch of the MSCEIT for inclusion in the final MATRICS Consensus Cognitive Battery (MCCB) [13]. The MCCB, including the social cognition domain, has since been used extensively [14]. Whilst the Managing Emotions branch of the MSCEIT is widely used as part of the MCCB, there have been only two previous studies utilising the full MSCEIT battery in people with schizophrenia [15, 16]. Eack et al. [15] found that the MSCEIT has adequate internal consistency and reliability in people with schizophrenia. Kee et al. [16] reported that people with schizophrenia had lower total MSCEIT scores compared to controls, with significantly worse performance on the Identifying, Understanding, and Managing Emotions Branches of the MSCEIT.

Many people with schizophrenia experience difficulties in social functioning, including problems with independent living, employment, and interpersonal relationships [17]. Whilst negative symptoms and cognitive impairment have long been known to correlate with social outcomes, these associations are modest and do not explain all of the difficulties in social functioning evident in schizophrenia [18]. Recent research has addressed the possibility that impaired social cognition might also contribute to poor functional outcome. Brüne et al. [19] administered tests of general intelligence, executive function, mental state attribution, symptoms, and social behaviour to 38 people with schizophrenia. Mental state attribution scores accounted for almost half of the variance in social function. Similarly, a meta-analysis by Fett et al. [20] showed that social cognition accounted for 23% of the variance in functional outcome in schizophrenia. However, there has been less research specifically investigating the contribution of EI to social outcome. If EI is found to make a significant contribution to social function, interventions designed to remediate deficits in EI may improve outcomes for people with schizophrenia.

 Kee et al. [16] assessed 50 people with schizophrenia using the MSCEIT. They found modest correlations between all Branches of the MSCEIT and independent living/self-care; between Identifying Emotions (Branch 1) and work productivity, relationships with family and spouse, and psychosocial adjustment and between Understanding Emotions (Branch 3) and psychosocial adjustment. Eack et al. [15] reported a significant association between total MSCEIT scores and the major role functioning subscale of the Social Adjustment Scale in 64 people with schizophrenia, but otherwise there was little relationship between MSCEIT scores and objective measures of functional outcome. A subgroup of these subjects undertook two years of intensive therapy designed to improve social cognition, attention, and memory. Improvements in Managing Emotions (Branch 4) of the MSCEIT were associated with improvements in functional outcome [21]. Improved neurocognition, and in particular improved executive function, was found to mediate the effects of cognitive enhancement therapy on functioning.

We developed a pilot study which compared EI, measured using the MSCEIT Version 2.0, in 20 people with schizophrenia and 20 controls matched for age, gender, and years of education. We assessed neurocognition and social functioning in both groups, and investigated the relationships between EI, neurocognition, and social functioning. We expected that total EI scores, as well as subscale (Branch) scores, would be significantly lower in the schizophrenia group compared to the control group. Specifically, it was hypothesised that after controlling for neurocognition, a significant positive correlation would be observed between EI and social functioning in both the schizophrenia and control groups. Given that a relationship between the Managing Emotions Branch (Branch 4) of the MSCEIT and social function has been demonstrated in previous studies [13, 21] we hypothesised that this particular branch of the MSCEIT would be most strongly associated with social function in our subjects with schizophrenia.

## 2. Methods

### 2.1. Participants

Twenty people meeting the diagnostic criteria for schizophrenia (DSM-IV-TR) [22] were recruited from public mental health services across metropolitan Adelaide, South Australia. The schizophrenia group were outpatients, living in the community, taking maintenance antipsychotic medication, and considered clinically stable by their treating clinicians. Control subjects matched by age, gender, and years of education were recruited by radio advertising. Potential control subjects were assessed using the Mini International Neuropsychiatric Interview (M.I.N.I) [23] and excluded if any symptoms of psychosis were present.

All subjects spoke fluent English and were screened for current major depression, alcohol abuse and dependence, and substance use disorders by a trained clinician, using the M.I.N.I [23]. They were excluded if these disorders were present. Other exclusion criteria for both groups included intellectual disability or neurological conditions that could interfere with psychological or cognitive functioning.

All subjects gave written informed consent after the study had been fully explained. The study was reviewed and approved by the Central Northern Adelaide Health Service Ethics of Human Research Committee and the University of Adelaide, School of Psychology Human Ethics Subcommittee. This research was carried out in accordance with the latest version of the Declaration of Helsinki.

### 2.2. Measures

Participants attended individual testing sessions that lasted for two to three hours. Demographic data was collected and screening measures administered. Participants who were eligible to participate in the study completed the measures of EI, neurocognition, and social functioning.

#### 2.2.1. Emotional Intelligence Was Measured Using the MSCEIT V2.0 [8]

The MSCEIT is a self-administered test which consists of 8 tasks based on 141 items, with two tasks forming a single branch. Tasks range from identifying emotion in human faces to identifying strategies to manage emotions in social situations. The MSCEIT V2.0 provides an overall EI quotient and four subscale (Branch) scores.

1. Identifying Emotions (Branch 1) refers to an individual's ability to recognise how they and those around them are feeling [24]. This involves attending to and decoding emotional signals in other people's facial expressions, tone of voice, or posture as well as the ability to accurately identify emotions within oneself.
2. Using Emotions (Branch 2) includes an individual's knowledge of the link between emotions and thinking, and the ability to take feelings into account when reasoning and problem solving [24, 25].
3. Understanding Emotions (Branch 3) refers to an individual's capacity to analyse emotions, appreciate their probable trends over time, and to understand their outcomes [25]. This includes the ability to recognise groups of related emotional terms and to understand the antecedents of different emotions, for example, that irritation and frustration might lead to anger [24].
4. Managing Emotions (Branch 4) includes the knowledge that it is adaptive to regulate emotions to facilitate positive social interactions [24, 25].

There are two methods of scoring the MSCEIT V2.0 described in the literature. Responses can be scored according to either the general or the expert consensus method, where scores for each response are weighted according to either the proportion of a general population sample or the proportion of a panel of emotion experts that endorsed the same response [25]. For the current study, the expert consensus weighted scores were used to ensure that higher scores represented superior levels of EI [25].

#### 2.2.2. Premorbid Intelligence Was Estimated Using the National Adult Reading Test (NART) 

(See [26].)

#### 2.2.3. Current Neurocognitive Functioning Was Assessed Using the Brief Assessment of Cognition in Schizophrenia (BACS) [27, 28]

The BACS includes assessments of verbal memory and learning, working memory, motor function, verbal fluency, and executive function [27]. BACS scores were converted to *Z* scores for statistical analysis [28].

#### 2.2.4. Social Functioning Was Assessed Using the Social Functioning Scale (SFS) [29]

The SFS is a 79-item scale which measures seven major domains of functioning thought to be crucial for successful community living for individuals with schizophrenia. 

### 2.3. Statistical Analysis

Independent-samples *t*-tests were used to compare total EI scores, the scores on each Branch of the MSCEIT V2.0, and total BACS and SFS scores for the two groups. Pearson's bivariate correlations were used to determine the correlation between MSCEIT V2.0, BACS, and SFS scores for each group. 

Separate linear regression analyses were performed to examine the relationship between MSCEIT V2.0 and SFS scores in each study group, with BACS scores as a potential mediator variable. To examine the relationships between variables, the following regression analyses were conducted.

1. The potential mediator variable (BACS score) was regressed on the independent variable (MSCEIT V2.0 score);
2. the dependent variable (SFS score) was regressed on the independent variable (MSCEIT V2.0 score);
3. the dependent variable (SFS scores) was regressed on the potential mediator variable (BACS scores).

To further examine the relationship between EI and social functioning, multiple regressions were performed for each group. SFS scores were entered as the dependent variable in each analysis. To test whether any relationship between MSCEIT V2.0 and SFS scores was mediated by BACS scores, BACS scores were entered at Step 1 and total MSCEIT V2.0 scores were entered at Step 2. 

We found that the schizophrenia group had greatest impairment on the Understanding Emotions Branch of the MSCEIT V2.0. Exploratory post hoc analyses, using Pearson's bivariate correlations, were undertaken to evaluate the correlations between scores on the Understanding Emotions Branch of the MSCEIT V2.0 and total and subscale scores on the BACS and the SFS. We expected that in the schizophrenia group, lower Understanding Emotions (Branch 3) MSCEIT V2.0 scores would be associated with poorer cognitive and social functioning, with a *P* value of less than 0.01 accepted as significant. 

There was a significant difference in premorbid intelligence scores between groups; therefore, a post hoc ANCOVA was performed to determine differences in MSCEIT V2.0 scores between groups with NART scores as a covariate. 

## 3. Results

Demographic characteristics are described in Table 1. The control group scored significantly better than the schizophrenia group on the NART. 

Differences between the schizophrenia and the control group on measures of EI, neurocognition, and social function are presented in Table 2. The schizophrenia group had significantly lower scores than controls on all subscales of the MSCEIT V2.0. The greatest difference was found on Understanding Emotions (Branch 3). A post hoc ANCOVA with calculation of effect size showed that 12% of the difference in MSCEIT scores was explained by premorbid intelligence whilst 24% was explained by group (schizophrenia or control). 

The schizophrenia group also had significantly lower scores on all of the BACS subtests. Their scores were lower than controls on all components of the SFS, and these differences reached significance for five of the seven domains of social functioning. 

The schizophrenia group did not show any significant correlation between MSCEIT V2.0, BACS, and SFS total scores (Table 3). The Understanding Emotions Branch of the MSCEIT V2.0 was significantly correlated with the BACS Total *Z* score, SFS independent performance score and the total SFS score. Managing Emotions (Branch 4) correlated with the SFS Recreation score. In addition, the BACS Total *Z* score correlated with the SFS Interpersonal Communication score. 

In the control group, Using Emotions (Branch 2), Managing Emotions (Branch 4), and the total MSCEIT V2.0 score all correlated with the SFS withdrawal/social engagement subscale. Managing Emotions (Branch 4) also correlated with the total SFS score. 

Regression analyses for the schizophrenia group revealed that total MSCEIT V2.0 scores were not a significant predictor of total BACS scores (*b* = 0.26, *P* = 0.28), with total MSCEIT V2.0 scores accounting for just 6.6% of the variance in total BACS scores. In addition, neither total MSCEIT V2.0 scores (*b* = 0.18, *P* = 0.45) nor total BACS scores (*b* = 0.28, *P* = 0.23) were significant predictors of total SFS scores, with each explaining just 7.9% and 3.2% of the variance in social functioning, respectively. 

Together, the total BACS and MSCEIT V2.0 scores accounted for just 9.1% of the variance in the total SFS scores. In addition, once total BACS scores were statistically controlled for, total MSCEIT V2.0 scores accounted for just 1.2% of the variance in the total SFS scores (*b* = 0.12, *P* = 0.64). 

Similarly, in the control group, total MSCEIT V2.0 scores did not significantly predict total BACS scores (*b* = 0.17, *P* = 0.47), with the total MSCEIT V2.0 scores accounting for just 2.4% of the variance in total BACS scores. Total MSCEIT V2.0 scores did not predict total SFS scores (*b* = 0.41, *P* = 0.07), and similarly total BACS scores did not predict total SFS scores (*b* = 0.18, *P* = 0.45). Hierarchical analyses for the control group participants showed that the combined influence of total BACS and total MSCEIT V2.0 scores explained 18.3% of the variance in total SFS scores. Once total BACS scores were controlled for, total MSCEIT V2.0 scores accounted for 15.1% of the variance (*b* = 0.39, *P* = 0.10). 

Post hoc analysis showed that in the schizophrenia group, there was a positive correlation between the score for Understanding Emotions (Branch 3) of the MSCEIT V2.0 and the BACS verbal fluency score (*r* = 0.646, *P* = 0.002). There were no other significant correlations between scores on Understanding Emotions (Branch 3) of the MSCEIT V2.0 and BACS or SFS subtests scores, for either group.

## 4. Discussion

Our results replicate the findings of Kee et al. [16] in demonstrating that people with schizophrenia are impaired on all aspects of EI, compared to control subjects. Impairment was greatest for Understanding Emotions (Branch 3). Understanding Emotions is assessed using tasks which require the participant to imagine that a person may be experiencing a particular emotion, then to imagine that this emotion may evolve into another related emotion. This is consistent with the literature showing that people with schizophrenia have impaired performance on TOM tasks [2], which assess the ability to correctly infer the thoughts and feelings of other people.

We had expected that impaired EI would be associated with poorer cognitive function, and that both EI and neurocognition would predict social outcome. In the schizophrenia group, there was a correlation between Understanding Emotions (Branch 3), the total BACS score, and SFS scores, confirming that impairment in recognising and understanding the emotions of others, and being able to predict the likely course of these emotions in a given set of circumstances, is of particular importance in schizophrenia. The correlation between Understanding Emotions and the total BACS score may indicate that the capacity for abstract thought and prediction of complex emotions and behaviours, necessary for successful completion of TOM tasks, was lacking in those with more impaired cognitive function. In turn, lack of capacity for affective TOM (knowledge about other people's emotions) [30] and impaired executive function [31] are both linked to poor insight, which in turn tends to be associated with poor outcome [32].

Eack et al. [15] found a positive correlation between all four Branches of the MSCEIT and a composite neurocognitive score in people with schizophrenia. The composite neurocognitive score reflected a battery of tests including processing speed, working memory, verbal memory, and executive function. Improvements in neurocognition and in Managing Emotions (Branch 4) were associated with better functional outcome [31]. Similarly, we found that in the schizophrenia group that the Managing Emotions Branch of the MSCEIT correlated with the SFS Recreation score. Associations between EI and social function were stronger in the control group, where Using Emotions (Branch 2), Managing Emotions (Branch 4), and the total MSCEIT score all correlated with social function.

Fett et al. [20], in a meta-analysis of the associations between neurocognition, social cognition, and community functioning in schizophrenia, reported that an overall neurocognitive factor accounted for only 6% of the variance in community functioning. In the present study, the MSCEIT did not significantly predict neurocognition or social functioning individually and neurocognition and EI together accounted for only 9.1% of the variance in social functioning scores in the schizophrenia group. These findings suggest that whilst neurocognition and social cognition do make some contribution to outcome, there are other factors which have a more substantial impact. 

We found that the associations between EI and social outcome were stronger in the control group than the schizophrenia group. This may reflect the presence of other factors specific to schizophrenia, such as poor insight or negative symptoms, modifying social outcome in the control group. 

This study was limited by the small sample size, and the groups were not well matched for premorbid intelligence, thus the study may have been underpowered. However, our within group analyses, looking at associations between EI, neurocognition, and social functioning, were not affected by this potential confounding factor. The correlational design of the study means the direction of the relationship between variables cannot be accurately deduced. For example, in the control group where a significant correlation between EI and social functioning was found, it may be the case that poorer social functioning leads to lower levels of EI, as lack of social contact results in fewer opportunities for individuals to learn social norms and gain experience in understanding the emotional reactions in others. 

## 5. Conclusion

EI has proven to be a very useful construct in the general population, and further research into EI could contribute to our understanding of the nature of deficits in social cognition in schizophrenia. Our findings provide a basis for further studies exploring the relationships between EI, general intelligence, the various domains of cognitive function, and functional outcome in schizophrenia. A deeper understanding of the scope and clinical correlations of social cognitive deficits may help to identify targets of new treatments to improve emotional processing and functional outcome. 

## Figures and Tables

**Table 1:** Participant demographic and clinical characteristics.

Variable	Schizophrenia		Controls		*t*	Cohen's *d*	*P*
	*N*	Mean (SD)	*N*	Mean (SD)			
Age	20	43.25 (9.15)	20	38.60 (10.86)	1.47	0.46	0.15
NART score	20	107.45 (6.90)	20	114.00 (4.30)	−3.82	1.14	<0.001***
Age of illness onset	19	23.63 (7.52)					
Duration of illness (years)	19	19.42 (9.90)					
Education (years)	20	12.50 (1.99)	20	14.80 (2.50)	−3.22	1.02	0.19

	*N*	%	*N*	%		*χ* ^2^	*P*

Gender							
Male	10	50	10	50			
Female	10	50	10	50			
Marital status							
Married	3	15	9	45			
In relationship	3	15	4	20			
Single	14	70	7	35		5.48	0.07

***Indicates *P* < 0.001.

**Table 2:** MSCEIT, BACS, and SFS scores for schizophrenia and control groups.

	Schizophrenia mean (SD)	Control mean (SD)	t	95% CI	Cohen's d	P
MSCEIT scores						
Branch 1—Perceiving emotions	98.98 (15.57)	110.98 (14.16)	−2.55	−21.53, −2.48	0.81	0.02*
Branch 2—Using emotions	96.14 (14.53)	107.50 (15.79)	−2.37	−21.07, −1.64	0.75	0.02*
Branch 3—Understanding emotions	81.80 (11.63)	105.70 (12.59)	−6.23	−31.66, −16.14	1.97	<0.001***
Branch 4—Managing emotions	90.75 (12.41)	101.06 (14.74)	−2.39	−19.03, −1.59	0.76	0.02*
MSCEIT global score	86.15 (11.81)	109.45 (14.46)	−5.58	−31.76, −14.85	1.77	<0.001***

BACS Scores						
Verbal memory	−2.16 (1.39)	−0.20 (1.02)	−5.09	−2.75, −1.18	1.92	<0.001***
Digit sequencing	−0.41 (0.77)	0.54 (0.51)	−4.63	−1.37, −0.54	1.86	<0.001***
Token motor task	−1.21 (1.31)	−0.03 (0.68)	−3.58	−1.86, −0.51	1.74	0.001*
Verbal fluency	−0.55 (0.97)	0.62 (0.69)	−4.41	−1.71, −0.63	1.70	<0.001***
Symbol coding	−0.88 (0.45)	0.45 (0.59)	−7.97	−1.66, −0.99	2.25	<0.001***
Tower of london	−0.49 (1.04)	0.61 (0.74)	−3.82	−1.68, −0.51	1.49	0.001*
BACS total *Z* score	−1.32 (0.93)	0.52 (0.66)	−5.09	−2.36, −1.32	2.28	<0.001***

SFS scores						
Withdrawal/social engagement	106.18 (11.60)	111.58 (12.41)	−1.42	−13.09, 2.29	0.44	0.163
Interpersonal communication	123.95 (19.56)	137.40 (12.40)	−2.60	−24.00, −2.90	1.08	0.014*
Independence performance	107.53 (7.70)	119.80 (7.53)	−5.10	−17.15, −7.40	1.63	<0.001***
Independence competence	111.85 (7.44)	121.13 (4.02)	−4.90	−13.14, −5.40	2.31	<0.001***
Recreation	100.39 (26.63)	124.00 (11.66)	−3.63	−36.77, −10.45	2.02	0.001**
Prosocial	101.13 (15.38)	114.28 (27.23)	−1.88	−27.31, 1.01	0.48	0.068
Employment/occupation	106.43 (15.34)	119.85 (3.79)	−3.80	−20.76, −6.09	3.54	0.001**
SFS total score	108.21 (8.03)	121.80 (6.57)	−5.86	−18.28, −8.89	1.85	<0.001***

*Indicates *P* < 0.05, **indicates *P* < 0.01, ***indicates *P* < 0.001.

**Table 3:** Bivariate correlations for MSCEIT, BACS, and SFS scores.

	Perceiving emotions	Using emotions	Understanding emotions	Managing emotions	Global EI score	BACS total *Z* score
	(Branch 1)	(Branch 2)	(Branch 3)	(Branch 4)		
Schizophrenia Group						
BACS total *Z* score	−0.06	−0.08	0.52*	0.14	0.26	
SFS Withdrawal/social engagement score	−0.09	−0.11	−0.03	0.01	−0.09	−0.09
SFS interpersonal communication score	−0.20	−0.21	0.18	0.03	−0.04	0.48**
SFS independence performance score	−0.08	−0.04	0.44*	0.14	0.16	−0.02
SFS independence competence Score	0.09	0.21	0.31	0.32	0.32	0.07
SFS recreation score	−0.40	−0.43	−0.10	−0.60**	0.54*	−0.14
SFS prosocial score	−0.35	0.05	0.41	0.17	0.05	0.36
SFS occupation/employment score	−0.38	−0.20	0.15	0.18	−0.13	0.25
SFS total score	−0.18	−0.03	0.48*	0.25	0.02	0.28
Control group						
BACS Total *Z* Score	−0.16	0.12	0.38	0.28	0.17	
SFS withdrawal/social engagement score	0.27	0.65**	0.42	0.50*	0.57**	0.15
SFS interpersonal communication score	−0.21	0.34	0.35	0.30	0.23	0.15
SFS independence performance score	−0.26	0.29	0.28	0.14	0.14	−0.11
SFS independence competence score	−0.14	0.21	0.28	0.02	0.06	−0.16
SFS recreation score	0.00	0.27	−0.02	0.18	0.12	−0.07
SFS prosocial score	0.00	0.32	0.28	0.31	0.28	0.34
SFS occupation/employment score	−0.27	−0.02	0.06	−0.04	−0.10	−0.10
SFS total score	0.01	0.53**	0.29	0.50*	0.41	0.18

*Indicates *P* < 0.05, **indicates *P* < 0.01.
